# Attitudes and perceptions of Australian pharmacy students towards Complementary and Alternative Medicine – a pilot study

**DOI:** 10.1186/1472-6882-8-2

**Published:** 2008-01-28

**Authors:** Evelin Tiralongo, Marianne Wallis

**Affiliations:** 1 School of Pharmacy, Griffith University, Gold Coast, Australia; 2 Griffith University Research Centre for Clinical Practice Innovation and Gold Coast Health Service District, Australia

## Abstract

**Background:**

With the increased usage of CAM worldwide comes the demand for its integration into health professional education. However, the incorporation of CAM into health professional curricula is handled quite differently by different institutions and countries. Furthermore, the evaluation of CAM curricula is complicated because students' ability to learn about CAM may be influenced by factors such as student's prior knowledge and motivation, together with the perceptions and attitudes of clinical preceptors.

The study aimed to describe the attitudes, perceptions and beliefs of second, third and fourth year pharmacy students towards complementary and alternative medicine (CAM) and to explore factors that might affect attitudes such as learning, preceptors and placements.

**Methods:**

Pharmacy students from a University in South East Queensland, Australia participated in the study. The study consisted of a cross-sectional survey (n = 110) and semi-structured interviews (n = 9).

**Results:**

The overall response rate for the survey was 75%, namely 50% (36/72) for second year, 77.3% (34/44) for third year and 97.6% (40/41) for fourth year students. Overall, 95.5% of pharmacy students believe that pharmacists should be able to advise patients about CAM and most (93.7%) have used CAM prior to course enrolment. Students' attitudes to CAM are influenced by the use of CAM by family, friends and self, CAM training, lecturers and to a lesser degree by preceptors. The majority of pharmacy students (89.2%) perceive education about CAM as a core and integral part of their professional degree and favour it over an additional postgraduate degree. However, they see a greater need for education in complementary medicines (such as herbal medicines, vitamins and minerals) than for education in complementary therapies (such as acupuncture, meditation and bio-magnetism). Knowledge and educational input rationalised rather than marginalised students' attitudes towards CAM.

**Conclusion:**

Pharmacy students perceive education about CAM as a core and integral part of their professional degree. Students' attitudes towards CAM can be influenced by learning, lecturers, preceptors and practice experience. The content and focus of CAM education has to be further investigated and tailored to meet the professional needs of our future health professionals.

## Background

Surveys of medical and pharmacy students report that the majority of students welcome the inclusion of CAM education in the medical [1-4] and pharmacy curricula [5,6]. However, most of the studies indicated that students had insufficient knowledge to be able to recommend or counsel about CAM [1,7]. Studies investigating the knowledge of pharmacy professionals also revealed that pharmacists generally rate their knowledge of CAM as inadequate and are not confident in answering patient enquiries [8-12].

A variety of CAM education strategies exist and tracking changes in learner attitudes is one strategy to document successful and effective CAM instruction. However, a perceived heterogeneity of baseline attitudes or beliefs towards CAM makes the evaluation of CAM curricula complicated [13]. Pharmacy students' ability to learn about CAM may be influenced not only by the available university education, but also by additional factors such as the student's prior CAM knowledge, the student's self motivation and perceptions of the role of the pharmacist and the attitude of placement preceptors and other pharmacists towards CAM, as well as the student's overall beliefs and attitudes [13].

A large amount of research has been undertaken to investigate the attitudes, beliefs and use of CAM by medical students worldwide [1,2,6,7,13-16], and to a lesser extent by pharmacy and nursing students [5,6,9,15,17,18] with only one of the studies [5] using semi-structured interviews in addition to survey methods for a more in-depth and detailed understanding of students' opinions. So far one study has reported the use and attitudes of Australian pharmacy students towards CAM [18] however the study did not include student interviews and did not look at the changes in students' attitudes and perceptions in relation to integrated CAM education throughout the pharmacy curriculum.

Generally, most studies reported a high self-use of CAM products (above 70%) by medical, pharmacy and nursing students [13,18]. The use of CAM seemed not to be largely influenced by family background [5], or friends and colleagues [7]. Some studies have reported gender differences among student use and attitudes, with female students having more positive attitudes towards CAM use and education [13,22], whereas other studies did not detect significant gender differences [5,18].

Although surveys with medical, nursing and pharmacy students reported that the lack of scientific evidence is the most significant barrier to CAM use for all students [1,6,9], the vast majority of students from all professional groups believe that CAM is based on ideas and methods from which conventional medicine can benefit and that CAM would play an important role in their future professional life [1,6]. Moreover, pharmacy, medical and nursing students alike perceived CAM as more useful after studying [6,15,17] and applying their knowledge [6].

Interestingly, a comparison of pharmacy students with nursing and medical students showed that pharmacy students were more skeptical about CAM with a significantly higher percentage believing that CAM treatments have no true effect on the treatment of symptoms, conditions and diseases; that CAM is a threat to public health [6] and that CAMs have only limited use [18]. In addition, training in herbal medicines and nutritional supplements was favored more by pharmacy students, while nursing and medical students wanted to acquire skills in complementary therapies such as chiropractic and massage [6,9].

The aims of this study were to describe pharmacy students' attitudes towards, beliefs about and perceptions of CAM and to compare these in three cohorts of pharmacy students (second, third and fourth year). By distinguishing between complementary medicines (CMs) and complementary therapies (CTs) this study also explored possible differences in attitudes, beliefs and needs for CAM education with regards to different CAM modalities.

## Methods

### Setting and sample

This study was conducted among pharmacy students at a publicly-funded university in South-East Queensland, Australia. The four and a half-year Bachelor of Pharmacy/Master of Pharmacy (BPharm/MPharm) curriculum in this university integrates evidence-based CAM education throughout the third, fourth and fifth year of the pharmacy curriculum. Specifically, an introductory module on the quality use of CAM focusing on CAM familiarization in the third year is followed by integrative and evidence-based CAM teaching to the fourth and fifth years incorporating CAM into clinical topics through lectures and clinical case studies. By the time the study was conducted the second year students had received only one CAM lecture with some information regarding the mechanism of interactions of drugs with the herbal medicine St John's Wort. The third year students had already attended a six-week introductory module on the quality use of CAM providing students with an overview of quality, safety and efficacy issues, as well as illustrating their overall role in the maintenance of health. The third year module lays the basis for evidence-based training by integrating CAM into the clinical subjects Pharmacotherapeutics 1, 2 and 3 that are taught in the fourth year and fifth year of the degree. CAM education is integrated into lectures and clinical cases educating pharmacy students about the management of specific disease states. At the time of the survey the fourth year students had already received one and a half semesters of training on evidence-based CAM therapy integrated into the clinical subjects

Students in the second, third and fourth years of the BPharm/MPharm degree program were invited to participate in the survey during lecture time. If they consented to participation they completed the anonymous survey and returned it to a sealed box prior to exiting the lecture hall. Students were asked at the end of the survey to indicate if they were interested in participating in an interview about the topic and if so to provide contact details. Ethical clearance for the study was obtained through the Human Research Ethics Committee of the institution.

### Data collection

Data were collected by cross-sectional survey and qualitative interviews. The questionnaire was administered following lectures in week 10 of the second semester in 2006.

#### Cross-sectional survey

The 50 item questionnaire consisted of five sections: 1) demographic information about students, 2) general attitudes towards and perceptions of CAM (amended from the literature [6,9,13]), 3) barriers to CAM use (questions used as stated in the literature [6,9]), 4) sources of information and knowledge about CAM (amended from the literature [9,18]) and 5) CAM knowledge.

The 15 attitude and perception items used a 5-point Likert rating scale and the10 knowledge items had possible responses of true, false and don't know. Other items were either multiple choice answers or lists were multiple options could be ticked.

#### Qualitative interviews

Semi-structured interviews (15–25 min) were conducted with nine students, three from each cohort, in weeks 11 and 12 of the second semester in 2006. The interviews took place in the School of Pharmacy and were audio taped. Specific interview questions such as: "Why are you interested/not interested in learning about CAM during your pharmacy degree?, Why do/don't you think that CAM will play a role in your professional life as a pharmacists?, Why do/don't you think that CAM can be useful in patient care?, Has your attitude towards CAM changed/not changed throughout your degree and if so why?" drove the beginning of the interviews. In a conversational style specific topics such as students' motivations and purposes related to studying CAM, their attitude changes towards CAM and their expectations of the curriculum were pursued.

Students were familiar with the distinction between CM and CT as it was explained and used in lectures and workshops. Moreover, a paragraph outlining the distinction between CM and CT was given at the start of the questionnaire and was read to the students at the start of each interview.

### Data analysis

Survey data analysis was entered into SPSS version14.0. Descriptive statistics such as frequencies, means standard deviations and ranges were used to summarise the data. For the Likert responses, all responses with any degree of agreement were grouped together as positive responses, and all responses with any degree of disagreement were grouped together as negative response. T tests and chi square were used to analyse differences between cohorts as appropriate. Results were considered significant when the *p *value was less than 0.05.

The transcripts from the interviews were independently coded and analysed by both authors, and were read and re-read while checking the tape recording for accuracy. Qualitative content analysis techniques were then employed [19]. The text was coded; each sentence was read and a label attached to the text. These codes were then grouped together into descriptive categories [19].

## Results

### Response rate

Of the 110 students participating in this part of the study 36 students were second year, 34 were third year and 40 were fourth year pharmacy students. The overall response rate for the survey was 75%, namely 50% (36/72) for second year, 77.3% (34/44) for third year and 97.6% (40/41) for fourth year students.

### Demographics

Differences in age and gender between the three cohorts did not reach statistical significance. Overall, 80.9% were equal or younger than 24 years of age, and 68.1% were female.

### Use/Background

The three cohorts of students had similar histories in terms of previous use of CAM. Overall 93.7% of students have used CMs and 38.7% have used CTs (Chi square = 1.7; p = 0.44; Chi square = 4.2; p = 0.12). Similarly, the family use of CMs and CTs did not reveal between group differences (Chi square = 0.6; p = 0.74; Chi square = 6.4; p = 0.04). Overall, 84.7% of students indicated that immediate family members such as partner, parents and siblings use CMs and 55% of them used CTs.

### Attitudes towards CAM

Nine attitude statements regarding CAM in general or CMs/CTs specifically were included in the questionnaire to which the students could either, strongly disagree or disagree, be neutral, or agree or strongly agree. Table 1 presents a summary of the total sample and a comparison of the proportions of each cohort who agreed with particular attitudinal statements

**Table 1:** Frequencies, percentages and Students' agreement with general CAM statements across the three cohorts

**Statements with which students agreed**	Total sample (n = 110) f (%)*	2^nd ^year (n = 36) f (%)*	3^rd ^year (n = 34) f (%)*	4^th ^year (n = 40) f (%)*	**p value^**
Clinical care should integrate the best of conventional and CAM practices.	99 (89.2)	33 (91.7)	31 (88.6)	35 (87.5)	0.835
CAM includes ideas and methods from which conventional medicine could benefit.	90 (81.1)	29 (80.6)	25 (71.4)	36 (90.0)	0.122
A number of CAM approaches hold promise for treatment of symptoms, conditions and/or diseases.	94 (85.5)	30 (83.3)	29 (85.3)	35 (87.5)	0.876
Treatment with CMs which are not tested in a scientifically recognised manner should be discouraged	61 (55.0)	20 (55.6)	21 (60)	20 (50)	0.683
Treatment with CTs which are not tested in a scientifically recognised manner should be discouraged	49 (44.1)	13 (36.1)	20 (57.1)	16 (40)	0.164
The results of CMs are in most cases due to a placebo effect.	12 (10.8)	0 (0.0)	11 (31.4)	1 (2.5)	< 0.0001
The results of CTs are in most cases due to a placebo effect	10 (9)	1 (2.8)	7 (20.0)	2 (5.0)	0.022
CMs are a threat to public health.	5 (4.5)	0 (0.0)	2 (5.7)	3 (7.5)	0.266
CTs are a threat to public health.	3 (2.7)	0 (0.0)	2 (5.7)	1 (2.5)	0.331

*Students who strongly agree/agree with the statement. ^Pearson Chi-Square

Overall the vast majority of the students saw the need for CAM integration in patient care (89.2%), thought that CAM included ideas and methods from which conventional medicine could benefit (81.1%) and believed that a number of CAM approaches hold promise for the treatment of symptoms, conditions and/or diseases (85.5%).

In contrast, only half of the students overall agreed with the statement that treatment with CMs or CTs which are not tested in a scientifically recognised manner should be discouraged. Less than 5% of the students agreed with the statement that CMs and CTs are a threat to public health. No significant differences between student cohorts could be detected for both statements. Significant differences (p < 0.0001 (CMs), p = 0.022 (CTs)) between cohorts were detected for the statements that the results of CMs and CTs are in most cases due to placebo effects. That is, 31.4 % and 20% of third year students agreed with the statements regarding CMs and CTs, respectively, whereas no more than 5% of the second and fourth year students agreed with these statements.

In the interviews students variously defined the role of CAM in patient care as using CAM as a first line option, a possibility in "minor ailments", "in most cases" or as a last resort. For example, one student stated,

*Depending on the situation and the illness they could probably be a good line of therapy (...). Some of these products have quite a good safety profile and a lot of history of use for those patients and it may be a good option for patients to use first and then if they don't have improvement then they would try a more conventional medication*.

Whereas another student thought that, *"If the medicine that was prescribed wasn't agreeing with them or didn't work, if they were terminally ill and nothing else would work or if it helped with side effects of other drugs, if they preferred natural medicines"*, then CAM should/could be recommended.

### Barriers

Quantitative data analysing the barriers to CAM use did not reveal differences between groups. Overall, students ranked the barriers in the following order: 1) lack of scientific evidence (86.5%), 2) lack of trained professionals (65.8%), 3) lack of government subsides (48.6%) and 4) concern about legal issues (27%).

When the students were asked in the interviews whether they would recommend CMs or CTs to patients, most students mentioned sufficient evidence as the prime prerequisite for any recommendation which is in line with the quantitative data. For example,

*If it's got strong evidence then I wouldn't hesitate in recommending it but if the evidence is lacking or a bit iffy (...) then I'd be on the side of caution*...

### Attitudes towards learning about CAM

The percentage of students agreeing with the statement that pharmacists should be able to advise on CMs increased with increasing years of study, with 100 % of the fourth year students agreeing with the statement. However, the difference between the three cohorts was not significant. Overall 95.5% of students were of the strong opinion that pharmacists should be able to advise patients about CMs (Chi square = 3.2; p = 0.2), but overall only 65.8% of students from all three cohorts were of the same opinion regarding CTs (Chi square = 0.85; p = 0.654).

Qualitative data indicated that students perceive learning about CAM as important because:

• Pharmacists sell CAM products.

• CAM knowledge is necessary to be a well-rounded professional.

• A pharmacist needs to have an evidence-based approach.

• CAM can be effective so as a pharmacist one need to know what is effective.

For example, one student stated that,

*It's out there in the pharmacy and people ask us about it so we need to know. (...) there is no one else really to go to apart from the health food shop, (...). We are the experts on drugs so we should know about it*.

Another student stated: *"(...) it's for sale in the pharmacy and people ask about it." *and "*Customers might think that you really trying to help them, like actually care about them."*The reasoning for the students also focused on the necessary evaluation of evidence *"...It would be good to be able to give evidence-based information to customers so they can understand and also to be able to determine between products that are good and products that don't have evidence."*

### Attitudes towards the integration of CAM education into the pharmacy curriculum

There were no significant differences between cohorts in their desire to learn about CMs and CTs during their university degree. Overall 89.2% of students strongly agreed/agreed that they wanted to learn about CMs and 74.8% (Chi square = 0.9; p = 0.64) wanted to learn about CTs (Chi square = 2.5; p = 0.28) during their university degree.

Students interviewed favoured the integration of CAM education into the pharmacy curriculum over an additional postgraduate degree. The interviews identified several reasons such as:

• CAM is part of pharmacy so it should be in the primary degree

• Would know about CAM when start working

• Would not necessarily do a post-grad degree on CAM

• University is an un-biased information provider

One student stated for example:

*It should definitely be included. (...) People expect you to know about the drugs, complementary medicines included. Not everyone is going to go on to do a postgraduate degree, but they are still going to be quizzed about the medicines*.

Moreover, one student said: "*I would much rather learn at Uni (...) So when you start your first job you are not oblivious to complementary medicines (...), you already know about it and you can then be working*." Similarly, one student highlighted: "*(...) you've got to integrate it. I wasn't going to bother going and doing another post graduate course when you have already been at Uni this long*". One student pointed out that: "*It should be done in a university setting where it is more generalised, it's more specific and it's not biased towards any specific product*." However, some students preferred time limitations on the CAM content "*to just spark an interest*". For example: "*I think it shouldn't be a massive part of the pharmacy degree obviously, but I think that (...) we should get a good insight into it so that you know a little bit about it anyway*."

### Attitudes towards the content of CAM education

Our survey distinguished between CMs (e.g. herbal medicines, minerals, vitamins) and CTs (e.g. acupuncture, meditation), as pharmacy students may have distinct priorities and attitudes towards specific modalities of CAM. The findings highlight students' demands for sufficient CAM education during their pharmacy degree; however, students indicated a higher interest in CMs (overall 89.2%) than CTs (overall 74.8%).

Qualitative data show that there are several reasons students believed that learning about CMs was more important than learning about CTs. Reasons were:

• The role of a pharmacist is to advise on medicines rather than therapies

• Students are more interested in medicines

• CMs are more important as they can interact with other medicines

As one student said,

*I think the medicines are more important just because that is what we have in the shops and that's what people expect us to know about. I mean if they want to know about acupuncture they will generally ring up an acupuncture clinic and ask them about it*.

However, some students feel that additional knowledge about CTs would be helpful in patient care and would make them a better pharmacist,

*I think predominantly about medicine but I think it would be an advantage to learn about what therapies have to offer as well. I think it is important, you know things like with acupuncture with pain relief (...) if you are a pharmacist and you are continually dispensing pain relief medication it would be good to have some sort of knowledge about therapies as well so that you can give people alternatives*.

### Influences on attitudes

Quantitative and qualitative data indicate that students' attitudes towards CAM are influenced, to different degrees, by various factors such as self-use, family and friends, as well as learning, lecturers, preceptors and placements.

#### Family, friends and self-use

Qualitative data indicated that most students were strongly influenced by family, friends and self-experience. This experience and/or influence resulted in both, skeptical views on CAM such as, *"I come from a fairly medical family, my dad was a GP and my sister is a Physio and I have looked into them [CAM] a bit and they did not convince me," *and more positive attitudes towards CAM such as:

*I actually have a best friend who has been diagnosed with *(a medical condition) *and she has tried everything (...) and before that I guess I didn't really have much of an opinion about it [CAM] really, but (...) I've seen that she has gotten better so my opinion has gone up I guess*.

#### Learning, lecturers and preceptors

Table 2 summarises quantitative data obtained from students in third and fourth year who evaluated whether learning, lecturers and/or preceptors had given them a more positive attitude towards CAM.

As can be seen in Table 2, learning had given greater than 50% of the students a more positive attitude towards CMs, with the numbers significantly increased in fourth year (85%). Around 50% of students in both cohorts indicated that learning about CTs had positively influenced their attitudes to CTs, with the numbers significantly higher in the third year cohort (54.3%). However, qualitative data showed that learning about CAM not only influenced students' attitudes towards CAM in a positive way, but that it also rationalised student thinking. Being more informed about CAM meant that students:

**Table 2:** Students in 3^rd ^and 4^th ^year who evaluated if learning, preceptors and lecturers had given them a more positive attitude to CAM

**Attitude Changes**	**3^rd ^year (n = 3) f (%)**	**4^th ^year (n = 40) f (%)**	p value*
**Learning**			
Positive attitude to CM	22 (63.9)	34 (85.0)	0.08
Positive attitude to CT	19 (54.3)	27 (45.0)	0.04
**Lecturers**			
Positive attitude to CM	27 (77.1)	39 (97.5)	0.007
Positive attitude to CT	25 (71.4)	32 (80.0)	0.38
**Preceptor**			
Positive attitude to CM	NA	20 (50.0)	
Positive attitude to CT	NA	14 (35.0)	

*p value related to chi square

• Became more cautious if recommending CAM

• Realised the potential of evidence-based CAM in patient care

• Were able to interpret evidence-based information on CAM

For example, students with a sceptical view on CAM stated *"I thought they [CAMs] would be just junk until I learnt what they did and whenever they worked or if they didn't, or how you could use them." *Another student commented

*I didn't really have a lot of faith in them (CAM) because I had read...there was a lack of evidence but after doing this course, there was a lot of evidence presented to us that you couldn't really deny that they don't have a place in the health of patients. ...I want to know more now so I read stuff and get more up to date*.

In contrast, students who had been unconcerned about possible negative effects of CAM became more cautious when recommending CAM in practice,

*I grew up just thinking that they [CAM] are all natural (...) since Uni (...) a lot of what we learnt has sort of really hit home (...) some of them aren't as good as I thought they were (...) it hasn't turned me off them, just made me like more informed and aware of the goods and bad of it*.

In addition, students indicated that learning about CAM has given them "*a feel for integration" *in patient care and has encouraged them to gather more evidence, for example,

*I think it has changed, (...) my attitude toward them has changed because now I know how to find the evidence and I know how to read the evidence so ... that gives you more of a position to make up your mind on whether something works or doesn't*.

Lecturers influenced more than 70% of students positively towards CMs and CTs with 97.5% of the fourth year students stating that the lecturers influenced their attitude towards CMs in a positive way (Table 2). In contrast, only 50% and 35 % of students stated that they were positively influenced by preceptors towards CMs and CTs, respectively. Qualitative data show that a variety of reasons may be responsible for the overall diminished influence of preceptors and placements on students' attitude. These are:

• Limited knowledge of the preceptor and/or pharmacy staff about CAM

• Low engagement of preceptor and/or pharmacy staff with CAM

• CMs and conventional medicines are separated in the pharmacy

For example:

*One of my preceptors for the placement, he was kind of interesting cause he knew actually nothing about complementary medicines; was sort of almost anti complementary therapies and complementary medicines – I thought this is not the right way to be (...) if anything it made me even stronger. I think you look narrow minded to have that perspective and I think if a customer comes in and asks him about something and he goes I think that's rubbish they are just going to go somewhere else*...

Another student commented,

*When I worked at the pharmacy the only time they ever gave out complementary medicines is when a patient came in and asked for them, they never recommended them. I don't think that will influence how I behave as a pharmacist but I know that (...) maybe complementary medicines are not being used to their full potential in pharmacies at the moment*.

## Discussion

With regard to age and gender the sample accessed for this study was similar to pharmacy students investigated in another Australian study [18]. Not surprisingly, a high self use of CMs (93.7%), similar to that reported in the abovementioned study [18], was identified. However, the reported use was higher than for pharmacy students in the US [6], the UK [5] and Hong Kong [17] which is possibly due to different time frames and modalities of CAM use being investigated. The CAM use in our study, however, correlated well with the CAM use reported for medical students' worldwide [13,16]. It should be noted that the difference in the use of specific CAM modalities reported in previous studies [5,18] was also reflected in this study with CMs being used more than twice that of CTs (93.7% CMs *vs*. 38.7% CTs).

Again, similar to previous studies [6,9], the majority of students in our study displayed a positive belief in the usefulness of CAM. Moreover, students were conscious of the contemporary role of the pharmacist in patient care with 100% of fourth year students believing that pharmacists should be able to advise patients on CMs. Similar to the student perspective that "the practice of medicine will benefit from an integrative system [20]" students in our study acknowledged CAM as a therapy concept and strongly believed that it should be integrated in clinical care and that a number of CAM approaches hold promise for the treatment of symptoms, conditions and/or diseases. Furthermore, our qualitative data show that some students go as far as disagreeing with the way CAM products are displayed and offered as treatment options in current Australian pharmacy practice.

Therefore it is not surprising that pharmacy students participating in this study firmly believed that CAM education should be integrated in the existing pharmacy degree. Students strongly preferred the incorporation of CAM education into the undergraduate degree over additional postgraduate studies to enhance work readiness and to save themselves time and money.

Previous studies have shown a decline in interest towards CAM education with increasing years of study [21], or have reported that perceptions of CAM education are not affected by the years of study [22]. Moreover, studies have also reported that biomedical training and increased education in a medical school increases students' scepticism towards CAM and results in a decline in students' desire for CAM education [14,21]. Our quantitative data agree with Greenfield *et al*. [22] that perceptions about CAM education do not depend on the year of study however qualitative data suggest a more focused interest.

Similar to the findings of Hon *et al*. [17] and Baugniet, Boon & Ostbye [15], who reported that the educational exposure to CAM is correlated with the perceived usefulness of CAM, our study shows that learning, lecturers and to a lesser degree preceptors can alter attitudes towards CAM.

Our qualitative data show that learning about CAM rationalised student thinking toward CAM instead of marginalising it, thus developing a balanced, more pluralistic student view on systems of healthcare beyond the medical mainstream. Students with a more positive attitude to CAM at the start of their degree changed to a more careful assessment of CAM therapy, whereas students with a more negative attitude realised that some CAM therapies are based on significant evidence and are possibly beneficial in patient care.

In agreement with Lie *et al*., who showed that CAM education for students should focus on knowledge and skill acquisition rather than attempting to change their already positive mindset [17] our study showed that there is no need to focus on changing students' attitudes as CAM education will rationalise students' attitudes towards CAM. However, the variety of published findings on the matter indicates that changing students' attitudes through education, depends on *how *CAM education is presented.

With regards to CAM educational content our study confirmed previous findings that pharmacy students prefer a comprehensive education in CMs (e.g. herbal medicines, vitamins, minerals) over education in CTs (e.g. acupuncture, meditation and bio-magnetism) [6,9]. Interestingly, this correlates with a 50% higher self-use of CMs over CTs and a 30% higher us of CMs over CTs by family members. Moreover, at least 7.2% of students had qualifications in CMs, but not in CTs. The personal familiarity with CAM can be of great importance as it has been shown to be associated with higher knowledge scores [1,5] and the willingness to refer others to CAM [6,17].

## Conclusion

Pharmacy students commence pharmacy education with different attitudes towards CAM and most will have used CAM prior to course enrolment. They perceive education about CAM as a core and integral part of their professional degree. Students' attitudes towards CAM can be influenced by learning, lecturers, preceptors and practice experience. An integrated curriculum approach to CAM education for pharmacy students can stimulate rationalised rather than marginalised student thinking about CAM resulting in a balanced, pluralistic student view of systems of healthcare beyond the medical mainstream. The content and focus of CAM education has to be further investigated and tailored to meet the professional needs of our future health professionals.

## Competing interests

The author(s) declare that they have no competing interests.

## Authors' contributions

ET conceived of the study, participated in the design, data collection, analysis and writing. MW participated in the design of the study, data analysis and the writing of the article. Both authors read and approved the final manuscript.

## Pre-publication history

The pre-publication history for this paper can be accessed here:


